# Liver vitronectin release into the bloodstream increases due to reduced vagal muscarinic signaling after cerebral stroke in female mice

**DOI:** 10.14814/phy2.15301

**Published:** 2022-05-09

**Authors:** Matthew P. Keasey, Chiharu Lovins, Cuihong Jia, Theo Hagg

**Affiliations:** ^1^ Department of Biomedical Sciences Quillen College of Medicine East Tennessee State University Johnson City Tennessee United States

**Keywords:** blood protein, cholinergic, ischemic stroke, mice, vagus nerve, vitronectin

## Abstract

Vitronectin (VTN) is a glycoprotein enriched in the blood and activates integrin receptors. VTN blood levels increase only in female mice 24 h after an ischemic stroke and exacerbate brain injury through IL‐6‐driven inflammation, but the VTN induction mechanism is unknown. Here, a 30 min middle cerebral artery occlusion (MCAO) in female mice induced VTN protein in the liver (normally the main source) in concert with plasma VTN. Male mice were excluded as VTN is not induced after stroke. MCAO also increased plasma VTN levels after de novo expression of VTN in the liver of VTN^−/−^ female mice, using a hepatocyte‐specific (*SERPINA1*) promoter. MCAO did not affect SERPINA1 or VTN mRNA in the liver, brain, or several peripheral organs, or platelet VTN, compared to sham mice. Thus, hepatocytes are the source of stroke‐induced increases in plasma VTN, which is independent of transcription. The cholinergic innervation by the parasympathetic vagus nerve is a potential source of brain‐liver signaling after stroke. Right‐sided vagotomy at the cervical level led to increased plasma VTN levels, suggesting that VTN release is inhibited by vagal tone. Co‐culture of hepatocytes with cholinergic neurons or treatment with acetylcholine, but not noradrenaline (sympathetic transmitter), suppressed VTN expression. Hepatocytes have muscarinic receptors and the M1/M3 agonist bethanechol decreased VTN mRNA and protein release in vitro via M1 receptors. Finally, systemic bethanechol treatment blocked stroke‐induced plasma VTN. Thus, VTN translation and release are inhibited by muscarinic signaling from the vagus nerve and presents a novel target for lessening detrimental VTN expression.

## Graphical abstract

Keasey et al., show that an increase in plasma vitronectin after ischemic stroke in female mice is produced by release from the liver. They show that vitronectin production is inhibited by the vagus nerve through muscarinic M1/3 receptors and can be blocked by systemic treatment with bethanechol. These findings are relevant to diseases characterized by detrimental vitronectin levels.

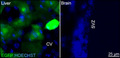

## 1 INTRODUCTION

Vitronectin (VTN) is a glycoprotein that binds to integrin receptors (Blouse et al., 2009; Mayasundari et al., 2004; Preissner, 1991; Schvartz et al., 1999) and is one of the most abundant proteins in blood (Alessi et al., 2011; Derer et al., 2009; Shaffer et al., 1984) where it is involved in hemostasis. VTN leaks into the brain after ischemic stroke (Jia et al., 2020; del Zoppo et al., 2012) possibly exacerbating the injury, which may relate to age‐related vascular and blood‐brain‐barrier dysfunction that worsens outcomes (Farrall & Wardlaw, 2009; Liu et al., 2009; Yamazaki et al., 2016). In mice, VTN is detrimental after ischemic stroke only in females (Jia, Malone, et al., 2020) by further increasing expression of the pro‐inflammatory cytokine IL‐6 (Keasey et al., 2018), inflammation (Milner et al., 2007) and tissue injury (Jia, Malone, et al., 2020) in the brain. Moreover, blood VTN levels increase only in female mice 24 h after an ischemic stroke and correlate with higher IL‐6 expression in the injured brain regions and greater injury sizes (Jia, Malone, et al., 2020). We therefore studied female mice, as male VTN is unaffected by MCAO. Outcomes after stroke are worse in women, especially after menopause (Ahnstedt et al., 2016; Bushnell et al., 2018; Kim et al., 2019). We wanted to define the unknown regulation mechanism of VTN induction after stroke in female mice as this might help to identify molecular targets to dampen the harmful increase.

VTN is primarily made in the liver and released into blood (Preissner, 1991; Preissner & Reuning, 2011; Schvartz et al., 1999). In the liver, VTN is produced by hepatocytes (Seiffert et al., 1991) and perivascular pericytes (stellate cells) (Jia et al., 2019). Little is known about the regulatory mechanisms under physiological or pathophysiological conditions. Blood IL‐6 levels increase after stroke in humans (Suzuki et al., 2009) and mice (Chapman et al., 2009; Tang et al., 2015), and systemic injection of IL‐6 can rapidly induce liver VTN expression in naïve rats (Seiffert et al., 1995). Another potential source of brain‐liver signaling after stroke is the cholinergic innervation of the liver by the parasympathetic vagus nerve (Lautt, 2009; Yi et al., 2010) where it has close interactions with pericytes (Jensen et al., 2013), Kupffer cells (Fonseca et al., 2019) and hepatocytes of the portal region (Lautt, 1983; Reilly et al., 1978). Hepatocytes have muscarinic cholinergic receptors (Li et al., 2009; Vatamaniuk et al., 2003). The female human (Abhishekh et al., 2013) and rat (Du et al., 1994) vagus nerve normally have a higher tone relative to males, and vagal brainstem nuclei have sexual dimorphisms (Akmayev et al., 1994; Ciriello, 2015; Dergacheva, 2015). Whether and how vagal tone may affect the expression of liver vitronectin has not been studied. Vagal dysfunction is thought to occur in many patients after stroke (Al‐Qudah et al., 2015; Mravec, 2010) and it is conceivable that the increase in blood VTN in female mice after stroke is caused by disinhibition due to reduced cholinergic signaling in the liver. The increased VTN‐driven inflammation seen in the female mouse injured brain would also be consistent with the findings that the vagus nerve has anti‐inflammatory activity in a number of disease models (Bonaz et al., 2016; Hoover, 2017), including stroke (Ay et al., 2016; Cai et al., 2014; Cheyuo et al., 2011).

Here, we determined whether the liver is the source of the increased plasma VTN after stroke in female mice and whether the regulation mechanism includes reduced vagus nerve transmission through muscarinic cholinergic receptors.

## 2 METHODS AND MATERIALS

### 2.1 Animals

A total of 145 female mice were used. VTN breeders (B6.129S2(D2)‐Vtn < tm1Dgi>/J, JAX 004371; backcrossed 13 times to C57BL/6) and C57BL/6 mice (JAX 000664) were from The Jackson Laboratory. All purchased mice were acclimated to our facility for at least one week before experiments. Mice were used at 10–12 weeks of age and weighed ~18–20 g. For all invasive procedures mice were anesthetized with Avertin given i.p. (400 mg/kg 2,2,2‐tribromoehtanol (Cat #T48402, Sigma) in 20 ml of 2% 2‐methyl‐2‐butanol in saline). Mice were placed on a 37°C water‐based heating blanket during the surgical procedures and their cages were placed on it for 7 d afterwards. Post‐operative care included subcutaneous injections of buprenorphine (0.1 ml of a 10 µg/ml solution, Covetrus, Cat #42023017905) to reduce pain, lactate ringers solution (2 ml), and gentamycin (0.1 ml of a 0.2 mg/ml solution). Mice were also given access to water saturated chow as well as bel vita breakfast cookies to assist and stimulate them to eat and reduce morbidity and increase survival. All animal work was approved by our University Committee on Animal Care and complied with the National Institutes of Health Guide on Care and Use of Animals.

### 2.2 Cervical vagotomy

A small incision was made in the neck along the ventral midline at the cervical level to expose the vagus nerves lateral to the left and right common carotid arteries by blunt dissection. Once exposed, the vagus was transected unilaterally using forceps before the closure of the surgical site. Mice were terminally anesthetized 24 h or 2 weeks later at which time whole blood was collected via the inferior vena cava and transcardial perfusion with ice‐cold PBS and isolation of liver, striatum, and subventricular zone for RNA and protein isolation.

### 2.3 MCAO stroke

Unilateral MCAO in mice was performed as previously described (Kang et al., 2013), reducing blood flow to the ipsilateral brain hemisphere below 20% of baseline as measured by Doppler flowmetry. Sham‐operated mice received the same surgery but without advancing the occluding filament. Alzet osmotic pumps (Cat #1003D, Alzet) were first primed overnight in saline at room temperature before filling with bethanechol (in saline and delivered at 20 mg/kg/day, Cat #23830, Cayman Chemicals). Osmotic pumps were placed subcutaneously via incision at the midline immediately after MCAO surgery. Tissues were collected 24 h after surgery or 28 days after surgery for mice maintained for behavioral and histological analysis.

### 2.4 Blood draw and plasma measurements

One day before and after cervical vagotomy or MCAO, mice were scruffed and blood was collected via cheek punch (~20–40 µl/draw; ~1–2% of total blood volume). A single drop of blood was collected in EDTA tubes (Cat #07 6011, Ram Scientific), centrifuged at 2000 g for 15 min before plasma supernatant was collected and stored at −80°C until used for measuring plasma levels of VTN. Plasma was diluted 1:10,000 and VTN concentration was determined by ELISA according to manufacturer's protocol (Molecular Innovation, Cat# MVNKT‐TOT‐1KIT). VTN KO plasma was used to confirm the specificity of the mouse VTN ELISA. For IL‐6 ELISA, whole blood was isolated and maintained at room temperature for 20 min to allow blood clotting. The clotted blood was centrifuged at 2000 g for 15 min at 4°C to isolate serum. ELISA was performed according to manufacturer's protocol with 50 µl/well (Cat # M600B, R&D Systems). Acetylcholine concentration was determined from undiluted serum according to manufacturer's protocol using 50 µl/well (Novus Bio Cat #NBP2‐66389).

### 2.5 Platelet isolation

Platelets were isolated from whole blood as described (Narciso & Nasimuzzaman, 2019). Briefly, 25 µl of 3.2% sodium citrate solution and 0.4 mM of Gly‐Pro‐Arg‐Pro (GPRP) was gently mixed with 200 µl of whole blood. Blood was then layered onto 600 µl of iohexol solution (12% iohexol powder in 0.85% sodium chloride, 5 mM Tricine, pH 7.2) in a polypropylene centrifuge tube. The sample was centrifuged at 400 g for 20 min at room temperature before the platelet layer was collected and transferred to a fresh centrifuge tube. Isolated platelets were diluted with 1 ml of PBS and centrifuged at 800 g for 10 min at room temperature. The resulting pellet was resuspended in 200 µl of PBS for VTN quantification.

### 2.6 Behavioral analysis

Motor function of the affected limb was assessed as before (Jia, Malone, et al., 2020) using a suspended unstable grid walk test performed before and at 7, 14, 21, and 28 days after MCAO. The number of missteps or footfalls with the affected forelimb were counted from videos as a percentage of the first 50 steps over a 90 s period.

### 2.7 Histological analysis

Brains were sectioned with a microtome and stained, with every sixth 30 µm section through the injury site labeled with antibodies against CD68 (activated microglia) or GFAP (astrocytes). Sections were imaged by mosaic stitching under a 10× objective. Inflammation was assessed by area and density of CD68 (1:500, Cat #MCA1957, Bio‐Rad, RRID_322219) staining while injury size was determined by a GFAP (Cat #MAB3402, Millipore, RRID_94844) negative area surrounded by GFAP positive astrocytes as described previously (Jia, Malone, et al., 2020). Both CD68 and GFAP were normalized to staining in the contralateral, non‐injured hemispheres using ImageJ software.

### 2.8 AML cell culture

AML12 cells are a hepatocyte cell line established from male CD1 mice and were obtained from ATCC (Cat# CRL‐2254) and maintained in DMEM/F12 medium (Cat #11320033, Thermo) supplemented with 1x ITS‐A (Invitrogen Cat 51300044), 40 ng/ml dexamethasone and 10% fetal bovine serum (Cat #16140, Thermo). At 24 h prior to experiments, AML cells were switched to a serum‐free formulation. SH‐SY5Y neuroblastoma cells are derived from human female metastatic bone tissue (Biedler et al., 1973) and were a gift from Dr. Meng‐Yang Zhu (ETSU). SH‐SY5Y cells were differentiated into cholinergic‐like cells by addition of retinoic acid (10 μM) to DMEM supplemented with 1% FBS and 2 mM L‐glutamine for 7 days. These cells were then split and plated in Neurobasal medium supplemented with B‐27 and retinoic acid as described (Shipley et al., 2016). AML cells were plated directly onto differentiated SHSY‐5Y cells for 24 h before RNA isolation and gene expression analysis by RT‐qPCR. Knockdown by siRNA was performed as previously described (Keasey et al., 2018) targeting M1 (Cat #L‐058643‐00‐0005, Horizon Discovery) and M3 (Cat # L‐042172‐00‐0005, Horizon Discovery) receptors or non‐targeting controls (D‐001810‐10‐05).

### 2.9 AAV virus preparation

Mouse cDNA was produced from reverse transcribed mouse mRNA (SuperScript Cat #18080051, Thermo). Mouse VTN was PCR amplified using the following primers: Forward, CCCAAGCTTAGAATGGCACCCCTGAGGCCC; Reverse, CGGGAATTCTTATGCCTTGTCGTCATCGTCTTTGTAGTCCTTCTCAGAGGTCGGGCAGCC. Reverse primer contained a FLAG tag sequence. Purified VTN cDNA was directionally cloned into HINDIII and ECORI at the 5’ and 3’ sites respectively into an AAV cassette (kindly provided by Dr. Ian Alexander) downstream of a human alpha antitrypsin promoter to drive expression specifically in the liver. AAV vectors were packaged in HEK293 t cells plated at 8 × 10^6^ cells in 10 cm dishes transfected with 5 µg VTN plasmid, 5 µg pAAV 2:8 (Addgene, Cat# 112864), and 15 µg pAdDeltaF6 (Addgene, Cat# 112867) per dish using PEI (25 k Da) at a ratio of 3:1 µg DNA. The medium was replaced with fresh Advanced DMEM (Cat #12491015, Thermo) supplemented with 2 mM L‐Glutamine 24 h post‐transfection. Medium was collected and replaced 48 h post‐transfection and every 24 h thereafter up to 5 days after transfection. At 5 days, the medium was removed, cells transferred to fresh tubes, centrifuged at 1000 g, and cell pellets combined in 10 ml of ice‐cold PBS supplemented with 200 mM NaCl and 0.001% F68 (Invitrogen, Cat# 24040032). Cells were freeze thawed in a dry ice/ethanol bath 3x to lyse cells and centrifuged at 3200 g. The resulting supernatant was treated with 50 U/ml Benzonase (Cat #70746, Millipore) for 45 min at 37 °C to degrade DNA carry over from the packaging process. Medium was centrifuged at 4000 g to remove cell debris before 25 ml of 40% PEG 8000 supplemental with 1 M NaCl was added per 100 ml of medium. The mixture was vortexed and left overnight to precipitate viral particles, then centrifuged at 2800 g for 15 mins. The resulting pellets were combined and resuspended in 10 ml PBS with 200 mM NaCl, 0.001% F68 and treated with 50 U/ml Benzonase for 45 min at 37°C. Crude viral suspensions were purified using an Iodixonal gradient (60%, 40%, 25%, 15%) and ultracentrifugation (350,000 g for 90 mins) according to standard protocol. The 40% layer containing virus was extracted and iodixanol removed via buffer exchange with PBS 200 mM NaCl, F68 buffer. Viral titer was determined by qPCR according to manufacturer's protocol (ABMGOOD, Cat #G931). AAV was injected at 5 × 10^10^ vg/mouse (Cunningham & Alexander, 2019) into the intraperitoneal cavity of female VTN^−/−^ mice at 6 weeks of age. Control mice were injected with a GFP only containing virus.

### 2.10 Western blotting

Cells and tissues were lysed in RIPA buffer and blotted as previously described (Keasey et al., 2013). Antibodies used were against beta‐actin (1:5000 #4970, RRID:AB_2223172), GAPDH (Cat #5174S, Cell Signaling, RRID_10622023), pFAK (1:1000 Cat #3283, Cell Signaling RRID AB_2173659), total FAK (1:1000 Cat #3285, Cell Signaling, RRID AB_2269034) or VTN (Cat #AB62769, Abcam, RRID_956454). VTN antibody specificity was confirmed in western blots using VTN^−/−^ liver (AAV‐GFP in Figure 2e).

### 2.11 RNA‐Isolation and RT‐qPCR

RNA was isolated according to kit manufacturer's protocol (Cat #R6834, Omega Biotek) and reverse transcribed is described previously (Keasey et al., 2018). RT‐qPCR was performed using TaqMan assays from Thermo against VTN (assay ID# Mm00495976_m1), SERPINA1A (Mm02748447_g1), IL‐6 (Mm00446190_m1), TNF (Mm00443258_m1) or IL‐10 (Mm00439616_m1), with GAPDH as internal normalizing control (Mm99999915_g1).

### 2.12 Statistical analysis

Statistical analysis was performed using Graphpad Prism (Version #8) software with One‐Way ANOVA with Bonferroni, or Dunnet post hoc tests, Two‐Way ANOVA or Student *t*‐test as indicated. Power analysis was performed using G*Power software (Version 3.1.9.3). For qPCR, ELISA, and western blotting, a minimum 3–4 experiments were required to obtain statistical significance based on a standard deviation of 10–20%.

## 3 RESULTS

### 3.1 Stroke causes an increase in plasma and liver VTN protein

**FIGURE 1:**
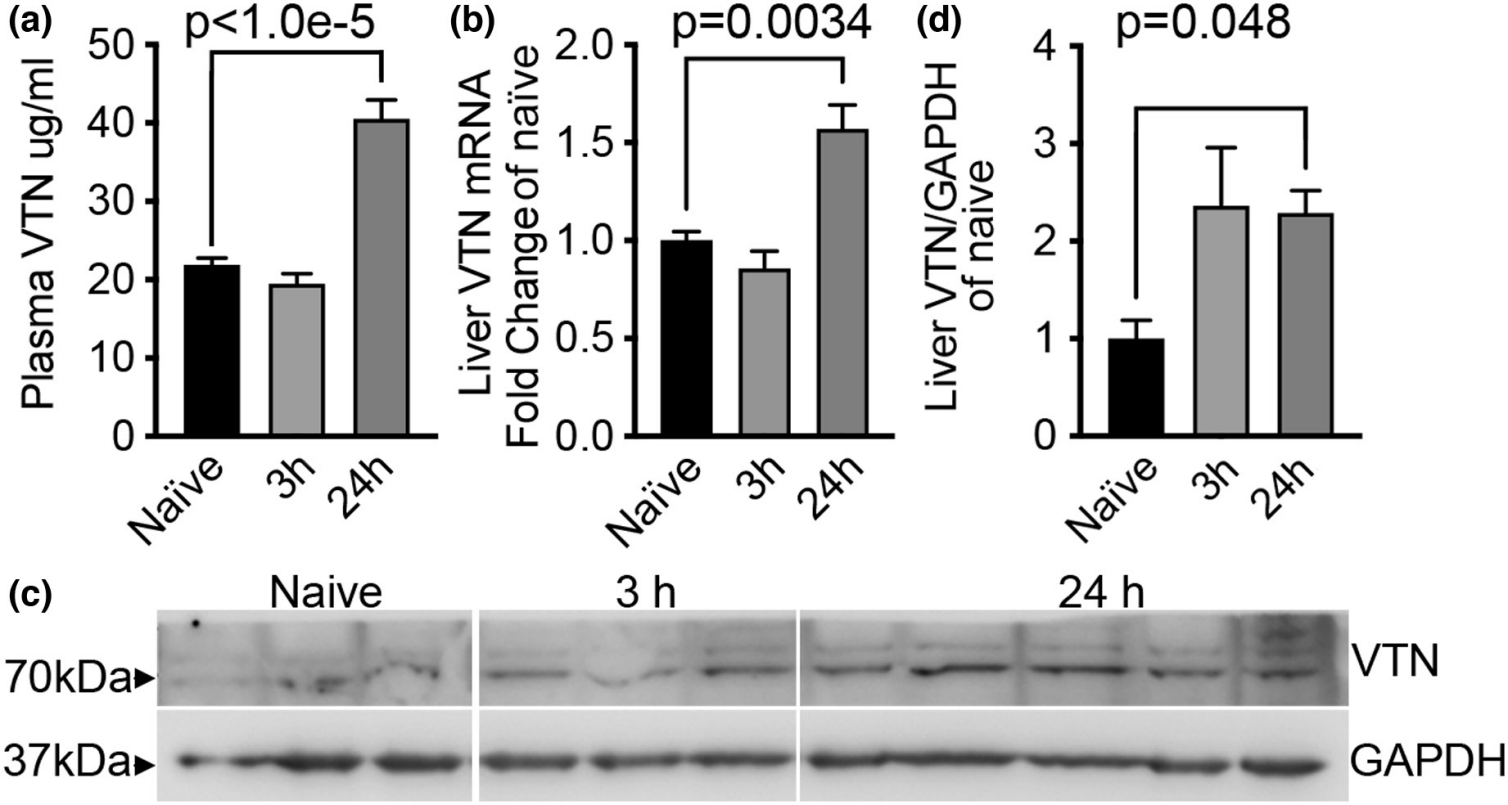
Plasma and liver VTN protein expression increases after stroke. (a) A 30 min ischemic stroke by MCAO in female C57BL/6 mice caused an increase in plasma VTN by 24 h, but not 3 h, after cessation of the stroke relative to naïve uninjured mice as measured by ELISA. Data = mean + SEM, *n* = 3,3,5. *p < 0.05, **p < 0.01, ****p < 0.0001 by ANOVA. (b) VTN mRNA levels in the liver were induced at 24 h, but not at 3 h, after MCAO relative to VTN levels in the livers of uninjured mice as measured by qPCR. Data are expressed as fold of naïve. (c) VTN protein expression in the liver was also increased at 24 h as shown by western blots of individual mice and quantified by densitometry of the VTN band as a ratio of the GAPDH loading control (d)

Adult female C57BL/6 mice with a 30 min MCAO ischemic stroke showed increases relative to naïve females in blood plasma VTN protein levels at 24 h, but not 3 h, after the cessation of the MCAO (Figure 1a). VTN mRNA expression was increased in the liver at 24 h post MCAO (Figure 1b), which was identified as the primary source of blood vitronectin by others (Seiffert et al., 1995). VTN protein levels were also increased at 24 h in the liver, as shown by Western blot (Figure 1c) and densitometry (Figure 1d).

### 3.2 Stroke increases plasma VTN released by the liver

**FIGURE 2:**
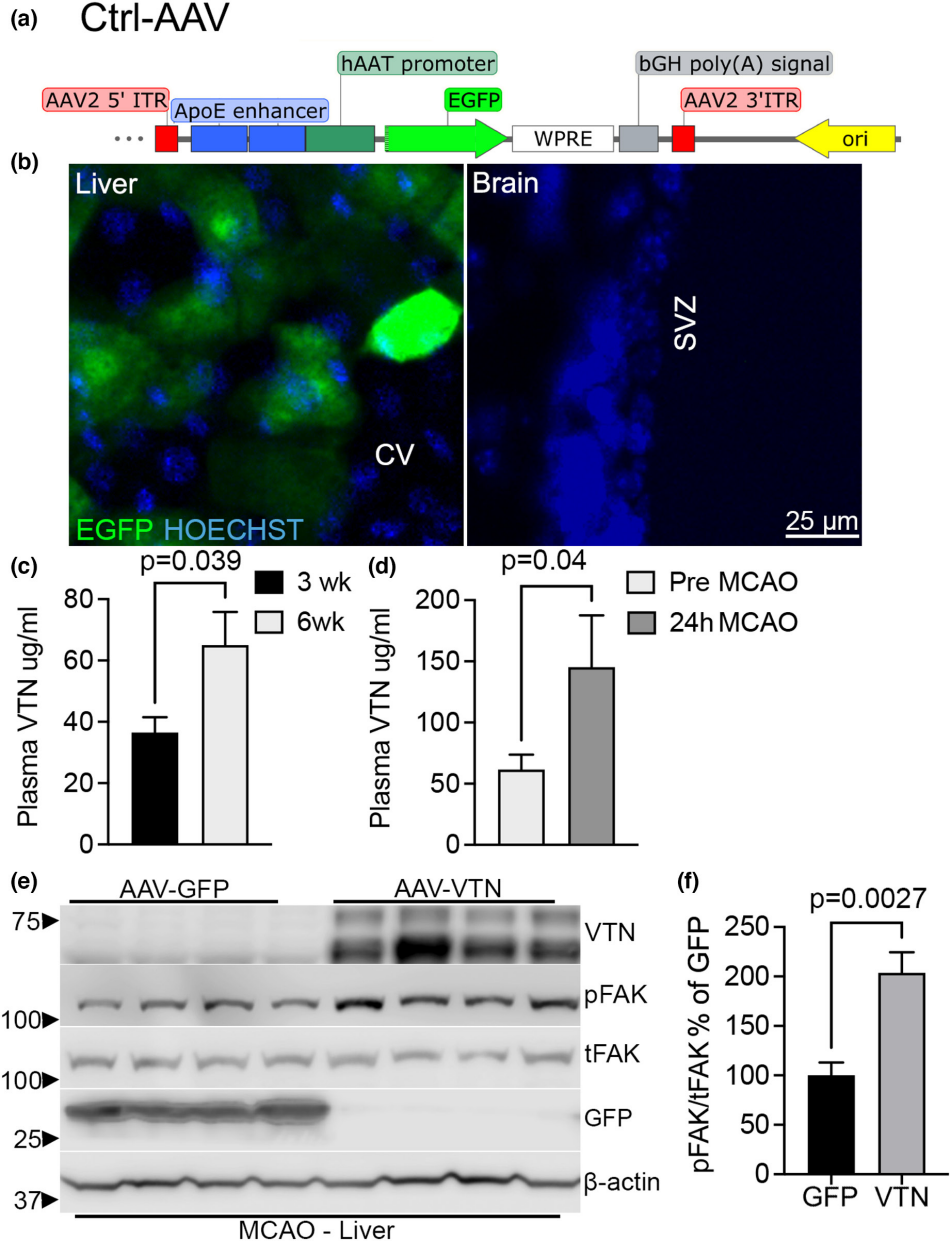
Plasma and liver VTN expression increase after stroke in VTN^−/−^ female mice with restored liver VTN. (a) Mouse VTN or GFP (control) were cloned downstream of a hepatocyte specific alpha antitrypsin promoter (hAAT, Serpina A1) within an AAV backbone. (b) AAV‐GFP vectors were injected into the peritoneal cavity of VTN^−/−^ female mice. After 6 weeks, GFP expression showed strong fluorescence in liver, but not brain sections. (c) VTN^−/−^ female mice injected with AAV‐VTN under a hepatocyte‐specific Serpina 1A (Alpha antitrypsin; hAAT) promoter had VTN plasma levels at increasing concentrations over a 6‐week period to within physiological range, as determined by ELISA. An AAV‐GFP construct in VTN^−/−^ females was used as a control. (d) AAV‐VTN females had a substantial increase in plasma VTN levels at 24 h after MCAO compared to their baseline levels before the stroke, as shown by ELISA. (e) The newly expressed VTN activated FAK in the liver as shown by pFAK in female mice at 24 h after an MCAO, as compared to AAV‐GFP controls as shown by Western blotting followed by densitometry (f)

We next investigated whether MCAO‐induced plasma VTN expression is mediated solely by the liver, given that brain pericytes also express VTN (Jia et al., 2019). Forced overexpression of mouse VTN was achieved in VTN^−/−^ female mice by I.P. injection of an AAV‐VTN viral construct containing a hepatocyte‐specific Serpina 1A promoter (Figure 2a; Loiler et al., 2003). Liver‐specific expression was confirmed by documenting the presence of GFP fluorescence from the AAV‐GFP control in the liver, but not in the brain (Figure 2b). VTN protein was released in the blood and increased over several weeks to physiological levels (40–60 μg/ml, Jia, Malone, et al., 2020) at 6 week after AAV‐VTN infection, as shown by ELISA (Figure 2c). In AAV‐VTN infected female VTN^−/−^ mice, stroke caused a 3‐fold increase in plasma VTN levels at 24 h after MCAO, as shown by ELISA (Figure 2d). The VTN protein was biologically active, as shown by activation of FAK in the liver of AAV‐VTN infected mice (Figure 2e,f).

### 3.3 MCAO‐induced increase of plasma VTN is not dependent on liver transcription

**FIGURE 3:**
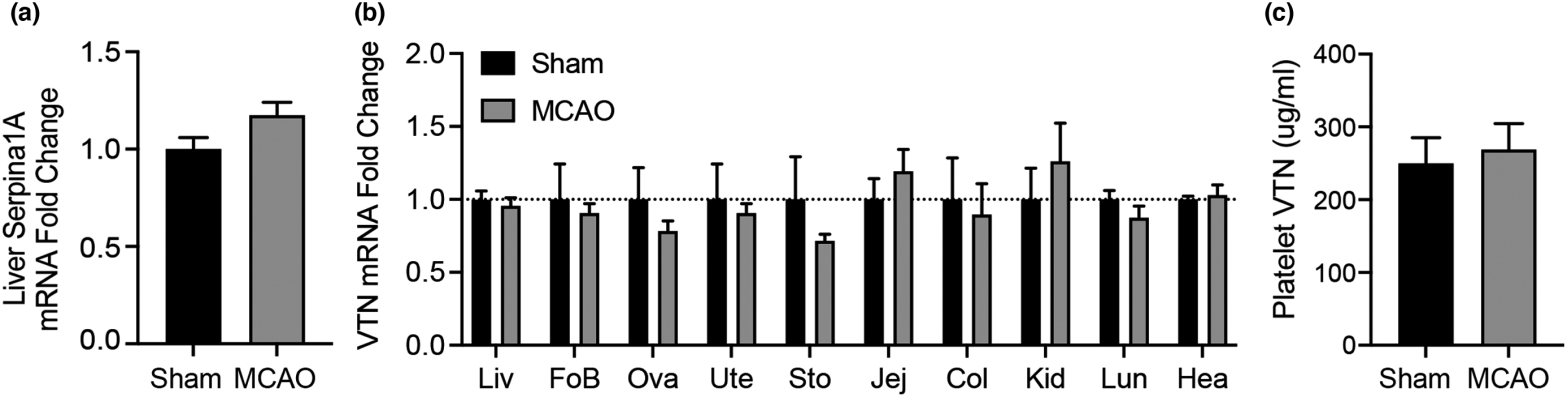
MCAO does not induce VTN mRNA relative to sham surgery. (a) At 24 h after MCAO, mRNA expression of the hepatocyte‐specific gene, Serpina 1A, in the liver was unchanged in the AAV‐VTN infected VTN^−/−^ mice. (b) In C57BL/6 mice, VTN mRNA expression has not induced the liver (Liv) or in other organs including forebrain (FoB), ovaries (Ova), uterus (Ute), stomach (Sto), jejunum (Jej), colon (Col), kidney (Kid), lung (Lun) and heart (Hea) at 24 h after MCAO relative to sham operated mice. (c) VTN protein content of platelets were not affected at 24 h post MCAO as measured by ELISA

The increase in VTN was not caused by mRNA upregulation because the level of liver Serpina 1A mRNA was not upregulated by the MCAO (Figure 3a) compared to sham operated females. Moreover, in the wildtype MCAO mice for which we reported increases in plasma VTN (Jia, Malone, et al., 2020), liver VTN mRNA was not different between sham and MCAO (Figure 3b). To rule out other sources for the increase in plasma VTN, we analyzed the forebrain where pericytes express VTN (Jia et al., 2019), but did not see an increase in mRNA. The female‐specific increase in plasma VTN (Jia, Malone, et al., 2020) suggested that VTN mRNA might be increased in female organs. However, VTN mRNA levels were not affected by the MCAO in the ovary or uterus which are known to express VTN (Carreiras et al., 1996; Seiffert et al., 1994). We also tested other organs known to produce VTN (Seiffert et al., 1991, 1994, 1996), but VTN mRNA was not affected by the stroke in the stomach, jejunum, colon, kidney, lung or heart (Figure 3b). Lastly, we excluded VTN release from platelets as a potential source for plasma VTN by finding no difference in MCAO relative sham mice (Figure 3c). Together, this suggests that VTN protein translation and/or release, and not transcription, is responsible for the stroke‐induced increases in liver and blood VTN in females.

### 3.4 The vagus nerve suppresses VTN release into the bloodstream

**FIGURE 4:**
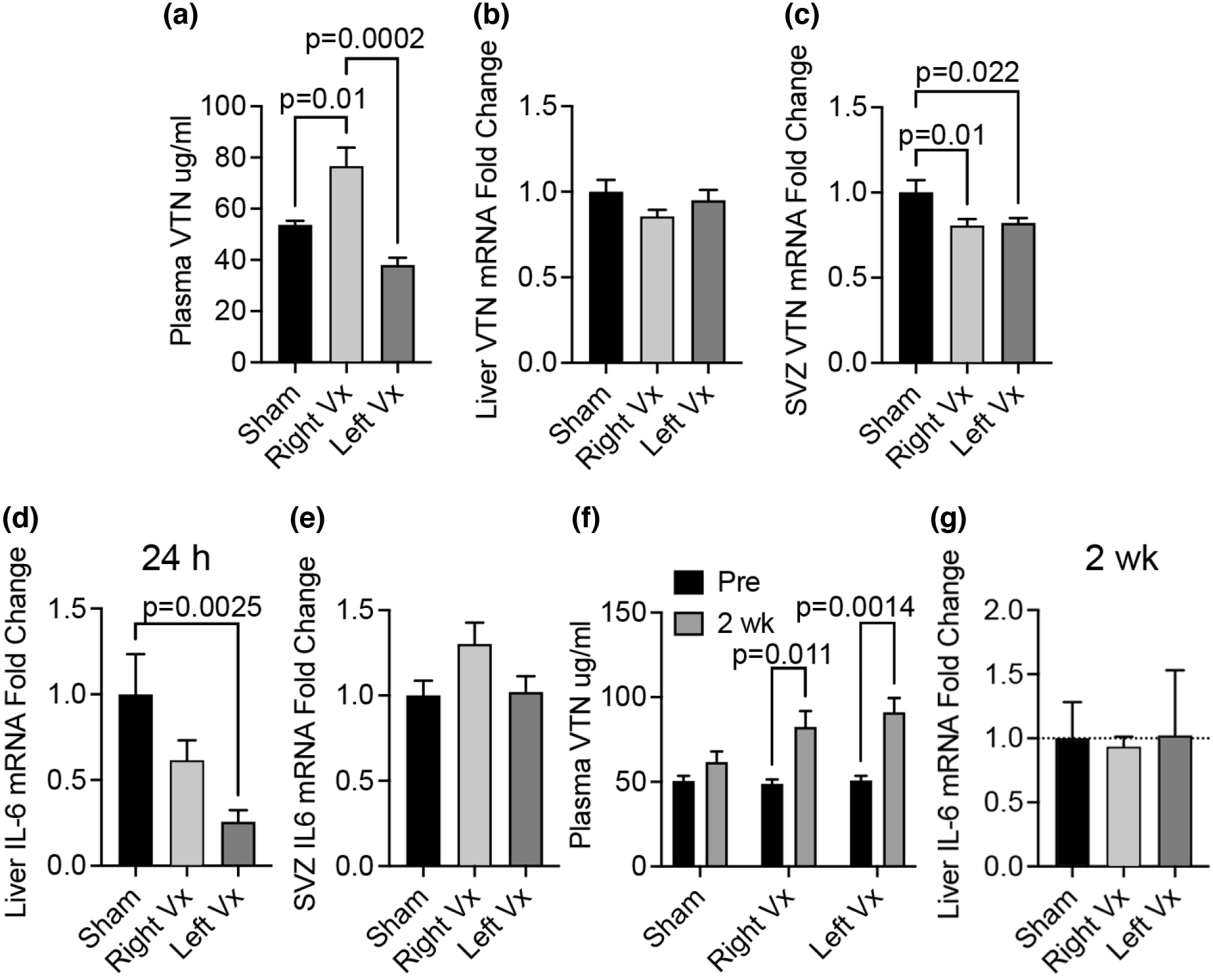
Vagotomy increases VTN release into the bloodstream. (a) Naïve wildtype C57BL/6 female mice with a unilateral right, but not left, vagotomy in the neck area have increased plasma VTN levels 24 h later, compared to sham operated females, as shown by ELISA. *N* = 5,5,5. *p < 0.05, **p < 0.01, ***p < 0.001 by ANOVA. (b) In these same mice, expression of liver VTN mRNA was not affected but was slightly reduced in the subventricular zone (SVZ) region of the brain in both left and right vagotomized mice relative to sham operated mice at 24 h (c). (d) IL‐6 expression was decreased by the left‐sided vagotomy. (e) No changes were observed in the medial striatum containing the SVZ area of the brain. (f) Two weeks after right or left vagotomy, plasma levels of VTN protein were increased compared to pre‐vagotomy levels as shown by ELISA. *N* = 5, 4, 5. (g) Liver IL‐6 mRNA expression was not affected

One way stroke in the brain could affect VTN release from the liver would be through changes in the vagal parasympathetic innervation. In naïve female C57BL/6 mice, transection (vagotomy) of the right, but not the left, vagus nerve in the neck area caused an increase in plasma VTN at 24 h compared to sham‐operated females (Figure 4a). This was not accompanied by changes in liver VTN mRNA expression (Figure 4b). We previously showed that VTN is also produced by pericytes in the brain (Jia et al., 2019) and we wanted to rule them out as the source of plasma VTN. VTN mRNA was decreased by ~20% in the medial striatum of the brain, which contains the subventricular zone, indicating that the brain is not responsible for the plasma VTN induction (Figure 4c). VTN can regulate IL‐6 expression in the brain (Jia, Malone, et al., 2020; Keasey et al., 2018). Moreover, injected IL‐6 can upregulate liver VTN (Seiffert et al., 1995). It was, therefore, important to document brain IL‐6 levels, expecting that VTN and IL‐6 levels would correlate. In contrast, IL‐6 mRNA was downregulated in the liver after cervical vagotomy mice relative to sham‐operated mice (Figure 4d). Expression of IL‐6 mRNA in the medial striatum was not altered (Figure 4e). Thus, IL‐6 is most likely not responsible for VTN plasma increases after vagotomy. This suggests that the vagus normally suppresses VTN release in the bloodstream and that right vagotomy has similar acute effects on plasma VTN levels as a stroke, which is known to cause reduced vagal tone in humans (Al‐Qudah et al., 2015; Mravec, 2010). To determine whether the vagus nerve is involved in chronic regulation of plasma VTN levels, they were measured at 2 wk after vagotomy. Plasma VTN levels were increased by both right and left vagotomy compared to sham, as shown by ELISA (Figure 4f). This suggests that baseline levels of plasma VTN are kept lower by innervation from both vagus nerves. Further, IL‐6 expression was not altered in the liver of these same mice (Figure 4g).

### 3.5 Acetylcholine suppresses VTN expression and release in vitro

**FIGURE 5:**
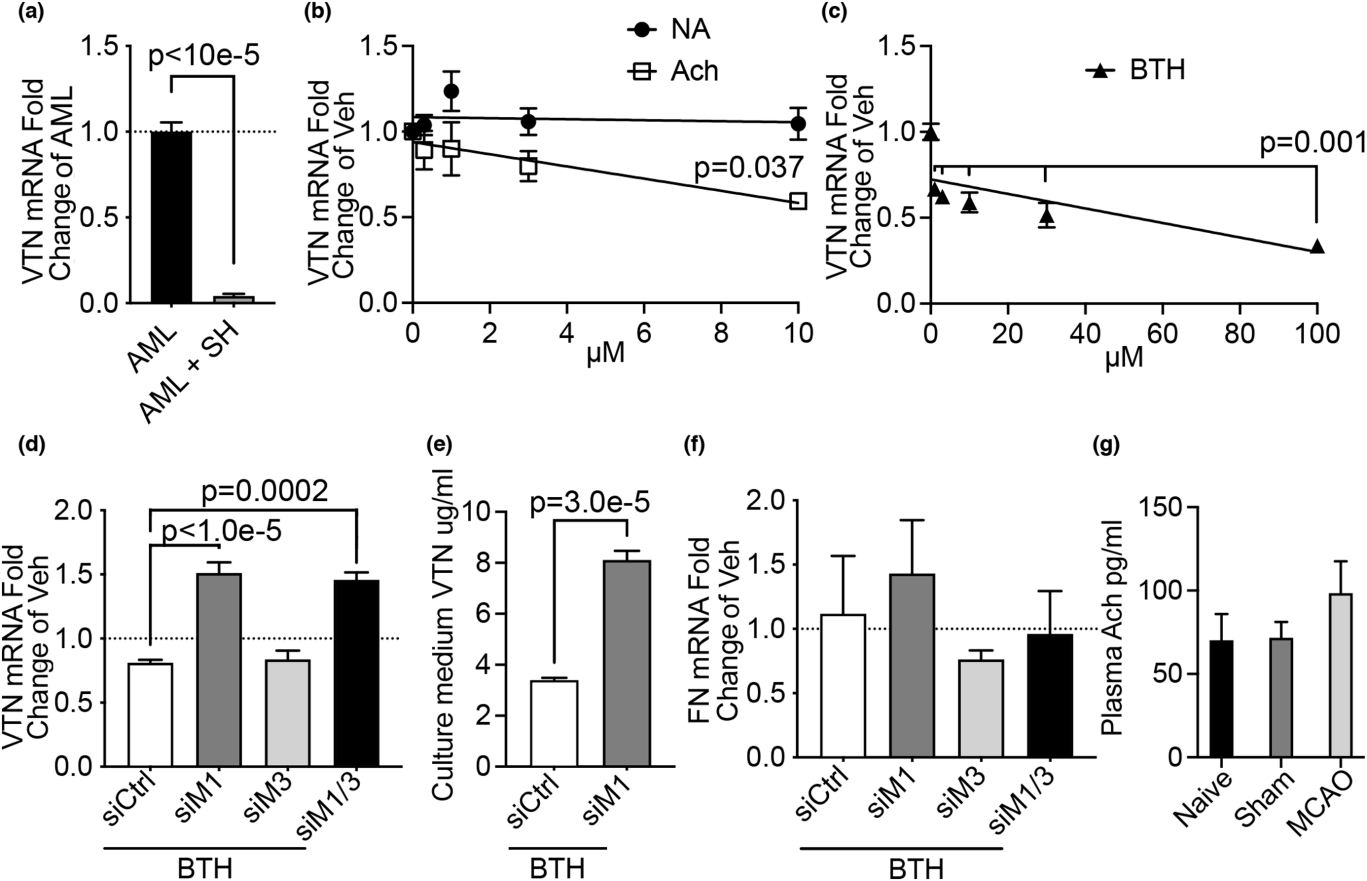
VTN expression is regulated by acetylcholine through muscarinic receptors. (a) Co‐cultured differentiated cholinergic SH‐SY neuronal cells had a large inhibitory effect on VTN mRNA expression by AML hepatocytes, as shown by specific qPCR for mouse VTN in AML and not the human SH‐SY cells (3 independent experiments each). (b) Acetylcholine (ACh), but not noradrenaline (NA), reduced VTN within 4 h in AML cells (*n* = 4 each concentration). (c) The M1/3 muscarinic agonist bethanechol (BTH) suppressed VTN expression with increased efficacy at higher concentrations (*n* = 3 per concentration). (d) SiRNA mediated knockdown of the M1 but not M3 muscarinic receptors in AML cells showed increased VTN mRNA under BTH conditions which suppress VTN expression (*n* = 4 each). (e) M1 siRNA increased the amount of VTN in the medium of AML cells in the presence of BTH (*n* = 4 each). (f) siRNA against M1 or M3 receptors had no effect on fibronectin (FN) mRNA expression in these BTH‐treated AML cells. (g) Acetylcholine levels in the plasma were not affected at 24 h after an MCAO compared to naïve or sham operated female mice. (*n* = 4, 4, 4)

The parasympathetic vagus nerve releases the neurotransmitter acetylcholine. In co‐cultures, differentiated cholinergic SH‐SY neurons almost completely blocked VTN mRNA expression in AML hepatocyte cells (Figure 5a). Acetylcholine, but not noradrenaline (produced by sympathetic liver innervation), reduced VTN expression in cultured alpha mouse liver 12 (AML) cells (Figure 5b). Mouse and rat hepatocytes are known to express M3 muscarinic receptors (Li et al., 2009; Vatamaniuk et al., 2003). Bethanechol, an M1/M3 agonist suppressed VTN expression, in a dose‐dependent manner, starting at 1 µM (Figure 5c). We defined whether M3 and/or the closely related M1 muscarinic receptors reduce VTN expression. Knockdown of M1 receptors, but not M3 receptors, in AML cells with siRNA had higher VTN expression than control siRNA, under conditions where bethanechol suppressed VTN (Figure 5d), suggesting that M1 receptors normally suppress VTN. The M1 muscarinic receptor knockdown also caused higher VTN protein release into the culture medium under bethanechol conditions (Figure 5e). The expression of fibronectin, another liver protein that is released in the blood, was not affected by either the M1 or M3 siRNAs under bethanechol conditions (Figure 5f). Finally, MCAO stroke did not cause changes in blood acetylcholine levels (Figure 5g), excluding the possibility that humoral cholinergic mechanisms cause the increase in plasma VTN. These data demonstrate that muscarinic signaling via M1 receptor regulates vitronectin expression in vitro, which helps explain the vagal effects because cholinergic transmission is the major way how the vagus regulates the liver.

### 3.6 Stroke induces VTN release into the bloodstream through M1/3 muscarinic receptors

**FIGURE 6:**
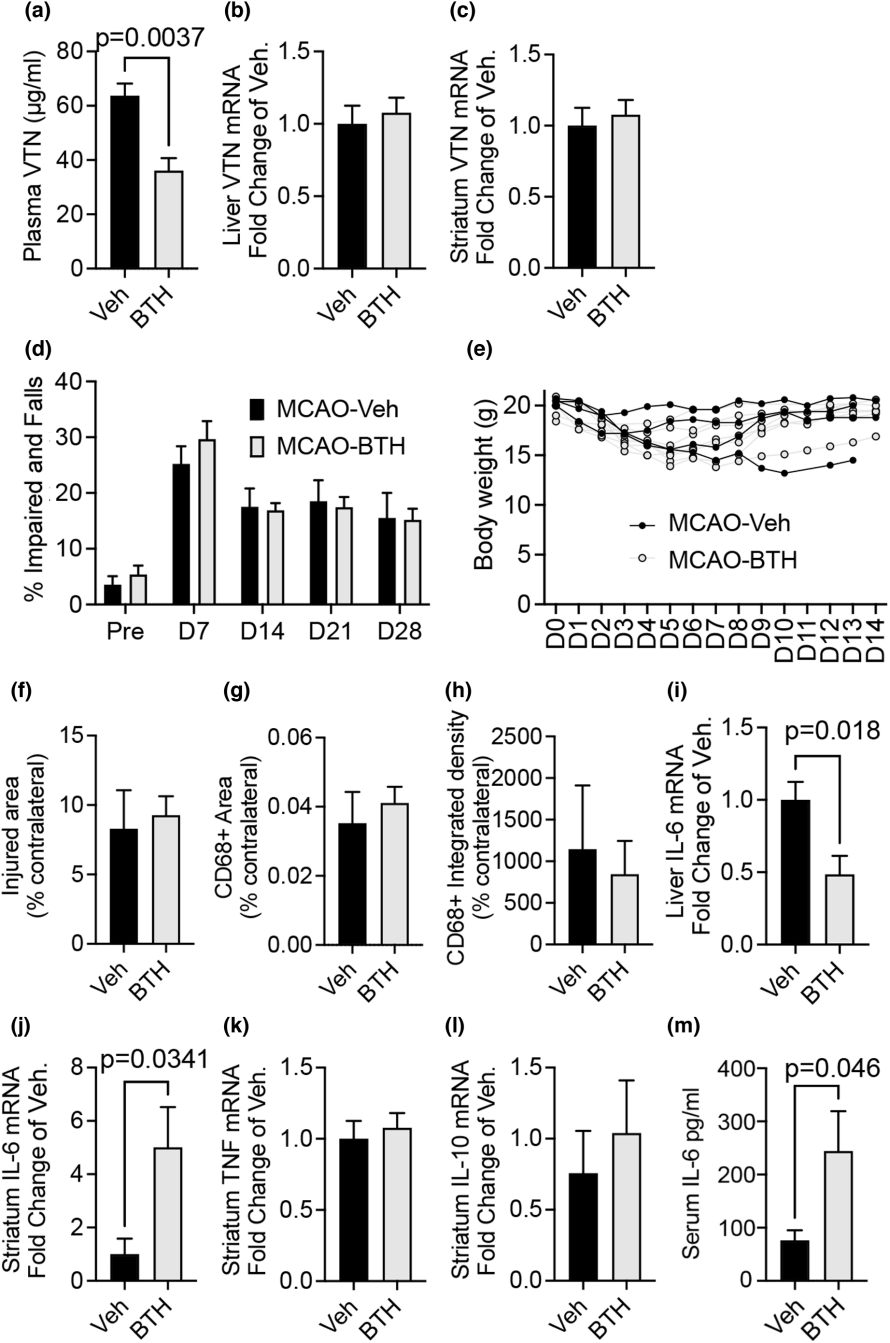
Bethanechol suppresses stroke induced plasma VTN but does not improve outcome. (a) Bethanechol (BTH, 20 mg/kg/day) was infused by Alzet osmotic pump placed subcutaneously for 24 h after an ischemic stroke by a 30 min MCAO in female C57BL/6 mice reduced VTN plasma levels at 24 h as shown by ELISA (*n* = 5,4). *p < 0.05, **p < 0.01, *t*‐test. (b) Liver VTN mRNA was not altered. (c) Similarly, striatal VTN mRNA was not significantly different after following BTH treatment relative to vehicle in MCAO mice. (d) Mice were infused with BTH (20 mg/kg/day) via osmotic pumps placed subcutaneously for 3 days post MCAO. Motor performance after stroke was not affected by BTH treatment, as measured by a grid‐walking test (*n* = 4,7). (e) Body weight was also not affected by BTH. (f) Histological assessment at 28 days after MCAO of tissue loss after stroke showed no effect of the earlier BTH treatment as measured in GFAP‐stained sections. (g, h) Inflammation was also not significantly affected by the earlier BTH treatment when measuring CD68 positive staining in injured brain tissue at 28 days. (9) At 24 h, liver IL‐6 mRNA was reduced in the liver or BTH treated MCAO mice in (A) relative to vehicle treated counterparts. (10) In contrast, striatal forebrain IL‐6, but not (11) TNF or (12) IL‐10, was robustly increased at 24 h after BTH treatment, as was (13) serum IL‐6 (*n* = 5,4)

We confirmed that the muscarinic mechanisms play a role after ischemic stroke. In adult female C57BL/6 mice, infusion of the M1/3 agonist bethanechol by osmotic pump (20 mg/kg/day), placed at the same time as MCAO reduced the plasma VTN levels seen at 24 h (Figure 6a). VTN mRNA was not altered in the liver (Figure 6b) nor striatum (Figure 6c) of MCAO mice treated with bethanechol. We tested whether bethanechol could be neuroprotective in female MCAO mice, as would be expected from the reduced plasma VTN levels at 24 h (Jia, Malone, et al., 2020). Female mice were infused with bethanechol (20 mg/kg/day) via intraperitoneal Alzet pumps for 3 days to target the 24 h peak of VTN which is normalized by 7 days, and the resulting IL‐6 induction, which is detrimental in females by causing excessive inflammation (Jia, Malone, et al., 2020) and avoid longer exposure to BTH. The bethanechol treatment had no effect on neurological function as tested in a sensitive grid walking test over a 4 wk period (Figure 6d), or on body weight (Figure 6e). At 28 days after MCAO, the extent of brain tissue injury was not different compared to vehicle‐treated females as measured in GFAP‐stained brain sections through the injury (Figure 6f). The injury size did not correlate with body weight (not shown). The area and intensity of microglial and macrophage activation, as an indicator of inflammation, was also not significantly different (Figure 6g,h). In the liver, IL‐6 mRNA levels were decreased at 24 h after MCAO in bethanechol relative to vehicle treated mice (Figure 6i). In contrast, and opposite to reduced plasma VTN, IL‐6 mRNA was upregulated in the injured striatum of bethanechol‐treated mice (Figure 6j). Pro‐inflammatory TNF (Figure 6k) and anti‐inflammatory IL‐10 (Figure 6l) were not affected by bethanechol. Plasma IL‐6 protein was also increased by bethanechol (Figure 6m). Together, this suggests that, although bethanechol reduced plasma VTN, which is expected to be beneficial after stroke in females, its side effects included increased detrimental IL‐6 expression in the injured striatum.

## 4 DISCUSSION

Our results suggest that hepatocytes in the liver are the source of increased detrimental blood VTN in female mice after ischemic cerebral stroke, and that the hepatic release of VTN is caused by reduced hepatocyte M1 muscarinic cholinergic receptor activation due to decreased vagal tone. We focused on female mice only, as male VTN is not altered at 24 h post MCAO and cannot rule out that similar VTN‐regulating mechanisms exist in males.

The liver has been considered the main source of VTN in the blood based on its high level of gene expression, especially in hepatocytes (Seiffert et al., 1991, 1995), whereas several other organs have much lower levels of expression. Perivascular pericytes in several organs, including the liver and the brain, also have high mRNA expression levels and much higher protein content than hepatocytes as shown by immunostaining (Jia et al., 2019). Thus, the increase in VTN after stroke in female mice could have come from peripheral sources. Our data showing stroke‐induced high plasma VTN levels in VTN^−/−^ females with VTN overexpressed only in hepatocytes suggest that hepatocytes in the liver are the sole sources of the increase in wildtype females. The finding that liver and plasma VTN protein increased in these mice after stroke without an increase in hepatocyte Serpina 1A mRNA in the liver is consistent with the finding that VTN mRNA in the liver of wildtype females is not increased either compared to shams. We excluded the possibility that the female‐specific VTN increase was due to increased gene expression in females or several other organs, including the brain. Thus, the increase in liver and plasma VTN protein after stroke is most likely caused by increased protein translation and release, respectively. Hepatocytes were known to express high levels of VTN mRNA while having small amounts of protein (Seiffert et al., 1991, 1994, 1996). Thus, VTN could be constantly released into the bloodstream, as suggested (Seiffert et al., 1995), and would explain the very high physiological concentrations in the blood. The combination of increased translation and release after stroke in females might constitute a highly regulable acute phase response mechanism that explains the very brief peak of increased VTN levels before they return to pre‐stroke levels within 7 days after stroke (Jia, Malone, et al., 2020). We find that VTN mRNA is increased in the livers of mice receiving an MCAO relative to non‐injured naïve controls but not compared to sham‐operated mice, despite plasma VTN being increased (Jia, Malone, et al., 2020; Figure 3d). This suggests that surgery may itself contribute to VTN mRNA changes but that VTN protein translation and/or release is dependent on the MCAO stroke. The intracellular mechanism that underpins this process remains to be elucidated. The differences in highly regulable VTN mRNA levels in cultured hepatocytes by cholinergic receptor activation and the very stable mRNA levels in the female mice after stroke or bethanechol treatment suggest that other mechanisms, including the cellular environment, regulate VTN mRNA expression in vivo (Jia, Malone, et al., 2020).

Our in vivo data suggest that the vagus nerve normally inhibits VTN release into the bloodstream and that its reduced activation of the liver after cerebral stroke causes the plasma VTN increase in female mice. Outcomes after stroke are worse in women, especially after menopause (Ahnstedt et al., 2016; Bushnell et al., 2018; Kim et al., 2019). In humans, stroke causes reduced vagal tone in many patients (Al‐Qudah et al., 2015; Mravec, 2010) but the mechanism is unknown. It will be important to determine whether sexually dimorphic vagal nerve activity plays a role in stroke and possibly other disorders with known increased plasma VTN levels (Anada et al., 2018; Derer et al., 2009). Vagal stimulation in male rats during the acute ischemic stroke phase reduces infarct size, inflammation, and blood‐brain‐barrier breakdown (Ay et al., 2016; Yang et al., 2018). It remains to be determined whether vagal stimulation would reduce VTN in female mice after stroke. In human stroke patients, transcutaneous vagal stimulators are used to improve motor rehabilitation during the chronic post‐injury phase (Dawson et al., 2016; Redgrave et al., 2018). Under normal physiological conditions, female mice have lower plasma VTN levels than males, which would be consistent with the findings that the vagus nerve normally has a higher tone in women (Abhishekh et al., 2013) and female rats (Du et al., 1994). The vagal brainstem nuclei have sexual dimorphisms (Akmayev et al., 1994; Ciriello, 2015; Dergacheva, 2015), including different urocortin‐1 positive innervation patterns (Ciriello, 2015). Urocortin‐1 injection can activate the vagus nerve (Chitravanshi et al., 2013) but it is unclear how this would be involved in regulating different baseline liver VTN expression levels in females and males. In addition, sexually dimorphic gene expression has also been found in the rat and mouse stellate ganglia which provide the main source of sympathetic innervation to the heart (Bayles et al., 2018, 2019). Our vagotomy data suggests that the right vagus regulates acute VTN release after stroke whereas both the right and left sides regulate the baseline level. The left subdiaphragmatic vagus forms the hepatic branch and the right the gastro‐duodenal branch (Yi et al., 2010). Vagal cholinergic innervation of the liver in rodents is sparse, especially compared to humans (Lautt, 2009). Cholinergic fibers have been found to course along the liver sinusoids adjacent to Kupffer cells, and that vagal stimulation regulates these resident macrophages during liver regeneration (Fonseca et al., 2019). Pericytes (Jensen et al., 2013) and hepatocytes (Lautt, 1983; Reilly et al., 1978) of the periportal region also are in close proximity to vagal endings. Thus, it remains to be determined which hepatocytes are involved in the VTN response after cerebral stroke and how acetylcholine reaches them.

The in vitro data showing acetylcholine inhibition of VTN synthesis and release by hepatocytes through M1 muscarinic receptors was confirmed in vivo by showing that the M1/3 agonist, bethanechol, reduced plasma VTN levels after the MCAO stroke. It remains to be determined whether muscarinic receptors in hepatocytes interact with known sexually dimorphic liver genes to explain the VTN release after stroke in females. Known genes include STAT5b which is lower in females (Holloway et al., 2007) and is a key regulator of sexually dimorphic genes in the liver (Oshida et al., 2016). STAT5b activity is regulated by growth hormone and the differences could be related to pulsatile versus chronic growth hormone release in females versus males. Finally, there are sex differences in the number and spatial distribution of the different cells of the liver (Marcos et al., 2016) but it is unclear how that would affect VTN release after stroke, specifically in females. It remains to be determined whether VTN is regulated in a mechanistically similar way in males. Since VTN is induced only in females at 24 h post MCAO, a female‐specific mechanism of gene regulation is evident. Perhaps, this occurs via lesser vagal effects in the liver, which remains to be determined by electrophysiological recordings and gain‐of‐function through vagal stimulation. Identifying this mechanism may go someway toward explaining sex differences after stroke.

The bethanechol treatment reduced plasma VTN at 24 h after the MCAO stroke in female mice, confirming that the in vitro data may explain our observations in vivo. Based on our findings that the VTN levels are predictive of the worse outcomes (Jia, Malone, et al., 2020) it was surprising that bethanechol was not neuroprotective. We chose bethanechol because it is safe for use in treating urinary retention problems and to stimulate saliva production in humans. However, bethanechol caused a large increase in IL‐6 expression in the injured striatum and plasma at 24 h after stroke in our mice. Pro‐inflammatory TNF and anti‐inflammatory IL‐10 were not affected, consistent with our findings that IL‐6 expression in the brain after stroke is particularly detrimental in female mice (Jia, Malone, et al., 2020). Thus, IL‐6 most likely counteracted the therapeutic effect of reducing plasma VTN. It remains to be determined whether we can target the VTN release mechanisms in the liver to reduce plasma VTN after stroke without increasing unwanted side‐effects.

In conclusion, we provide evidence in females that liver and plasma VTN protein levels are suppressed by M1 receptor cholinergic signaling on hepatocytes and that increased VTN expression in the blood after stroke is produced by the liver, most likely via vagal innervation. This is relevant for disorders such as stroke and cardiovascular disease, and also for disorders of the liver where VTN is a key marker for liver fibrosis and cirrhosis (Koukoulis et al., 2001).

## CONFLICT OF INTEREST

None.

## AUTHOR CONTRIBUTIONS

Matthew P. Keasey, Chiharu Lovins, and Theo Hagg designed research and analyzed the data. Theo Hagg and Matthew P. Keasey wrote the paper. Matthew P. Keasey and Chiharu Lovins performed experiments.
